# National Convention for the Revision of the Pharmacopœia

**Published:** 1850-02

**Authors:** 


### THE MEDICAL EXAMINER.

### PHILADELPHIA, FEBRUARY, 1850.

### NATIONAL CONVENTION FOR THE REVISION OF THE PHARMACOPŒIA.

The call for this Convention, to meet in the City of Washington on the first Monday in May, 1850, has been made by the proper authority, and we would call attention to the importance of an early appointment of Delegates by the bodies who have a right to representation. These bodies are "the several incorporated State Medical Societies, the incorporated Medical Colleges, the incorporated Colleges of Physicians and Surgeons, and the incorporated Colleges of Pharmacy, throughout the United States." The names of the Delegates appointed are to be sent officially to Dr. G. B. Wood, Philadelphia, Vice President of the last Convention. We hope that speedy action in the matter will be taken by the Societies, &c., interested.

